# Supplementary material for: Diversity spurs diversification in ecological communities
Source: Nat Commun. 2017 Jun 9;8:15810. doi: 10.1038/ncomms15810 (PMC5494188; doi:10.1038/ncomms15810)
Supplement: Supplementary Information [file ncomms15810-s1.pdf]

Type of file: PDF
Size of file: 0 KB
Title of file for HTML: Supplementary Information
Description: Supplementary Figures, Supplementary Tables, Supplementary Notes and Supplementary References.

Type of file: pdf
Size of file: 0 KB
Title of file for HTML: Peer Review File
Description:

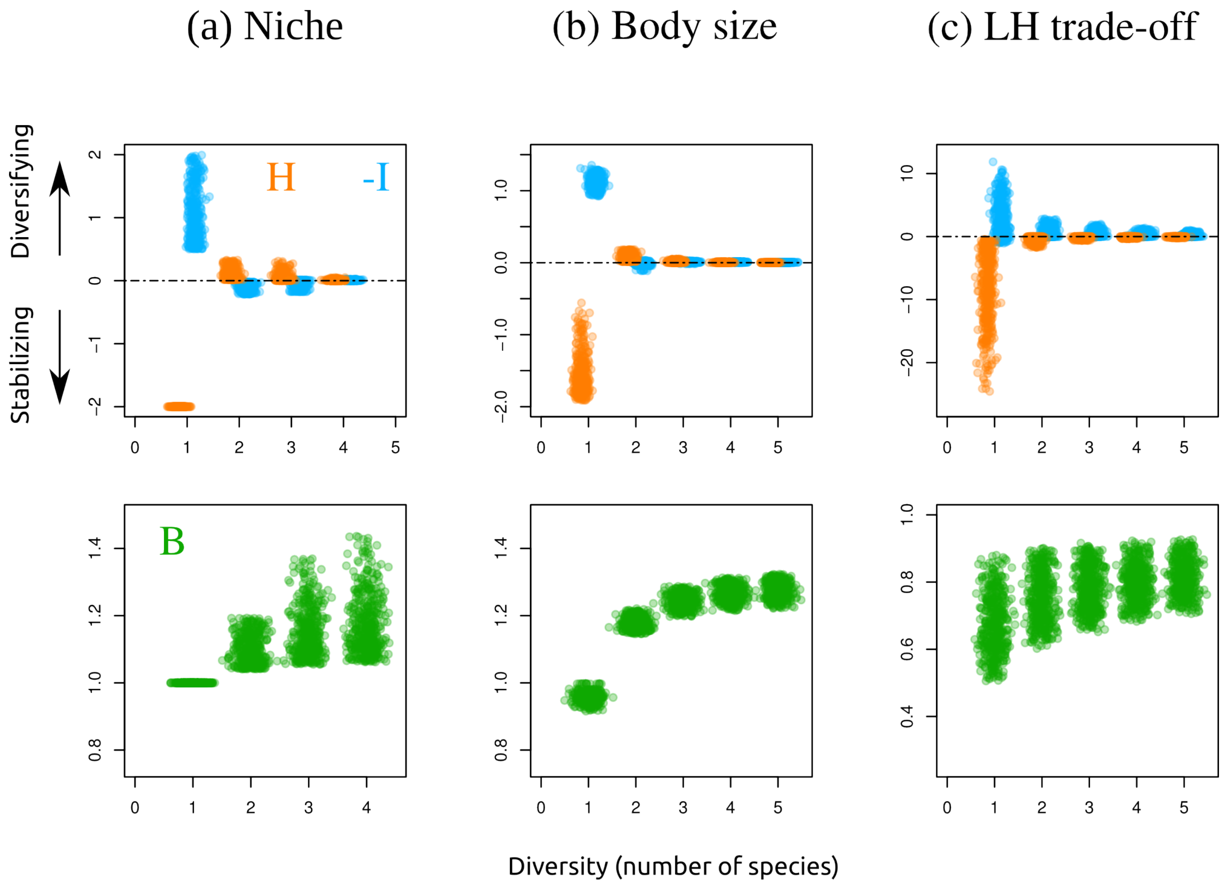

Supplementary Figure 1: Values of the three selection components, for each diversity level and each ecological scenario. Each time 500 dots are shown corresponding to 500 parameter combinations uniformly sampled over the parameter space explored by continuation. Dots were jittered horizontally. $-I_i$ was shown rather than I_i, to facilitate visualization, since positive values of H and negative values of I both favor diversification. Also, recall that greater values of B (green) mean an increased weight of the I (blue) component over the H (yellow) in determining the resulting type of selection (diversifying versus stabilizing).

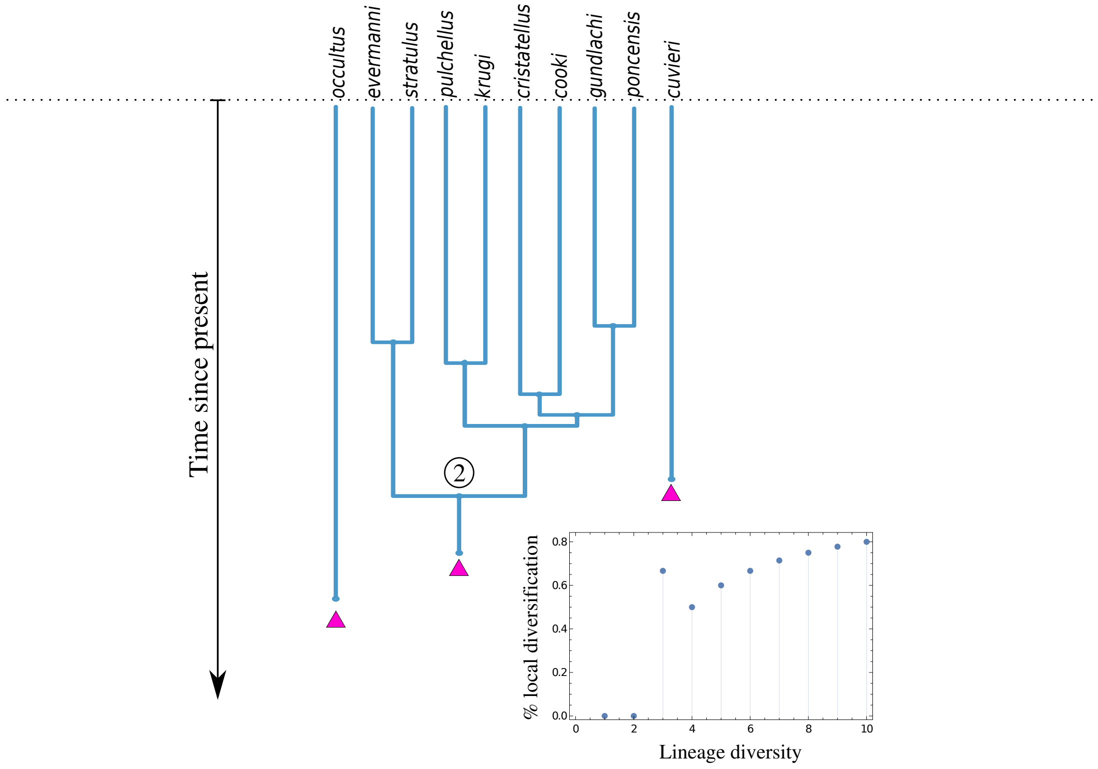

Supplementary Figure 2: An alternative scenario for the diversification of Anoles on Puerto Rico. According to the maximum clade credibility tree of earlier studies[1], adaptive radiation started after the second colonization event, and the *cuvieri* lineage colonized the island afterwards, which would mean a DDAR with minimal diversity of two rather than three. Data from[1].

Supplementary Note 1

We here provide details on the functions used to implement the three ecological scenarios in the generalized model.

Resource competition (niche)

The generalized equation (2) in the main text is most often used in its Lotka-Volterra form, to model competition along a resource gradient, such as a range of seed sizes for granivorous birds. In this case trait x represents the average position of a species along that gradient, or mean niche position (e.g. mean beak size). Different species might exploit different parts of the resource spectrum, according to classical ”niche partitioning”.

This scenario makes the following assumptions regarding the shapes of the functions: the carrying capacity function k, that directly reflects the distribution of the resource along the gradient, is usually assumed to be dome-shaped. This reflects the assumption that resources are most abundant for some intermediate type and are progressively scarcer as we deviate from it. Occasionally, k is taken to be constant over its entire range, i.e. $k(x) = k_0$ as in[2], but this yields degenerate evolutionary properties. The intrinsic growth rate r is typically taken to be constant and equal to one. Last, the competition function a should be an even function strictly decreasing in the trait difference $|x_i - x_j|$.

Early studies considered simple normal functions for both k and a, but it has become more and more clear that this choice entailed some non-robust and atypical properties, especially when there are multiple species. Alternative functions, sharing the same qualitative properties, have consequently been preferred recently, such as generalized normal functions. In our case, we also observed atypical properties of the normal/normal case. We thus considered different modifications to this original scenario, including generalized normal functions (from Gaussian to quartic), boosted normal functions (normal plus a constant), and Lorentzian functions. Since we obtained similar conclusions for these different cases, we present in the main article results for only one choice of functions that is simple and close to the original modelling assumptions. Specifically, we used a Lorentzian function for the carrying capacity:

$$k(x) = 1/\left(1 + x^2/s_k^2\right) \tag{1}$$

where parameter s_k controls the width (scale) of the resource distribution, just as it does in a usual normal function. This function has qualitatively the same shape as the classical normal function (Figure 1), and it yields exactly the same evolutionary behavior and branching criterion in the one-species case. We then used the classical assumptions for r and a:

$$r(x) = 1 \tag{2}$$

$$a(x_i, x_j) = \exp(-(x_i - x_j)^2/s_a^2) \tag{3}$$

where parameter s_a controls the width of the competition function (“niche width”). In Supplementary Note 3 we present a detailed evolutionary analysis of the niche scenario, where we consider an alternative choice of functions for k and a.

Parameters s_k and s_a were taken as our bifurcation parameters and varied in $(0.75, 1.25)$.

Asymmetric competition (body size)

An extension of the earlier niche model has often been used to model asymmetric competition between species[3;4]. Originally, this was introduced by including a skew parameter in the normal competition function, so that it is no longer symmetric, and can take values greater than one (thus representing competitive interference). The use of identical normal functions for k and a causes the same lack of robustness as in the symmetric model, and, as in the previous case, we considered different functional forms.

We chose to present here results using a a log-normal carrying capacity function:

$$k(x) = \exp(-\log(x)^2) \tag{4}$$

The log-normal distribution is the closest analogous to the normal distribution when one wants a distribution on positive reals, which is the case here, as body-size is a positive quantity.

We then used the classical assumptions for r and a:

$$r(x) = 1 \tag{5}$$

$$a(x_i, x_j) = \exp(d^2/s_a^2)\exp(-(x_i - x_j + d)^2/s_a^2) \tag{6}$$

where parameter s controls the width of the competition function ("niche width") and parameter d controls the level of competitive asymmetry.

Parameters s_a and d were taken as our bifurcation parameters and varied in $(1.2, 2.5)$ and $(0.08, 0.32)$, respectively.

Life-history trade-off (LH trade-off)

We used the standard patch-occupancy model describing the competition between species in a metacommunity, when there is a trade-off between colonization ability at the regional scale and competitive dominance at a local scale[5]. A general formulation of the model[6] is:

$$\frac{1}{n_i}\frac{dn_i}{dt} = x_i(N - \sum_j n_j) - \mu + x_i \sum_{j \neq i} \eta(x_i - x_j)n_j - \sum_{j \neq i} x_j n_j \eta(x_j - x_i) \tag{7}$$

Here n_i is the number of habitat patches or microsites occupied by species i, with at most one species occupying a site at any time (i.e. there is competitive exclusion at the local site scale). N is the total number of sites in the metacommunity. Trait x_i is the colonization rate of the species, and μ the rate of site destruction (or mortality), which for simplicity is taken to be species independent. Function η is the trade-off function that connects the difference in colonisation abilities between two species to the probability that the first species can displace the second from a given patch. This encapsulates the competitive asymmetry and also the level of habitat preemption[6]
.

This model is often scaled by taking dynamic variables to be fractions of occupied sites ($n' = n/N$) rather than absolute numbers. The same equations hold for n' if one rescales the colonization rates ($x' = x/N$) and the total number of sites ($N' = N/N = 1$).

Establishing an equivalence between equation (7) and a Lotka-Volterra form of equation (2) in the main article is straightforward. Grouping together density-independent and density-dependent terms yields:

$$\frac{1}{n_i}\frac{dn_i}{dt} = x_i N - \mu + \sum_j n_j \left[x_i \eta(x_i - x_j) - x_i - x_j \eta(x_j - x_i)\right] \tag{8}$$

Factoring out the density-independent terms:

$$\frac{1}{n_i}\frac{dn_i}{dt} = (x_i N - \mu)\left(1 + \sum_j n_j \left[\frac{x_i \eta(x_i - x_j) - x_i - x_j \eta(x_j - x_i)}{x_i N - \mu}\right]\right) \tag{9}$$

Now, the density-dependent effect of a species on itself is

$$x_i \eta(0) - x_i - x_i \eta(0) = -x_i \tag{10}$$

Dividing the ratio in square brackets by the previous quantity allows to isolate n_i:

$$\frac{1}{n_i}\frac{dn_i}{dt} = (x_i N - \mu)\left(1 - \frac{n_i + \sum_j n_j\left[1 + x_j\eta(x_j - x_i)/x_i - \eta(x_i - x_j)\right]}{N - \mu/x_i}\right) \tag{11}$$

This exhibits the equivalence with equation (1):

$$r(x) = xN - \mu \tag{12}$$

$$k(x) = N - \mu/x \tag{13}$$

$$a(x_i, x_j) = 1 + x_j\eta(x_j - x_i)/x_i - \eta(x_i - x_j) \tag{14}$$

and function g of Lotka-Volterra form.
The trade-off function η should be decreasing with $x_i - x_j$, and we used a standard logistic form[6]:

$$\eta(x_i - x_j) = \frac{\gamma}{1 + e^{\alpha(x_i - x_j)}} \tag{15}$$

Parameter α controls the intensity of the CC trade-off and parameter γ controls the level of competitive preemption[6]. We used these two parameters as our bifurcation parameters, and they were varied in $(2, 15)$ and $(0.4, 1)$, respectively. Other parameters were $N = 1$ (so that n is a fraction of occupied patches) and $\mu = 0.1$.

Remark that the competition function a, in contrast to the two previous scenarios, depends on x_i and x_j independently. In addition, it does not tend to zero as trait differences gets large. Indeed, $a(x_i, x_j) \to 0$ when $x_i \ll x_j$ but $a(x_i, x_j) \to 1 + x_j\gamma$ when $x_i \gg x_j$. This indicates that even species very distant in trait space can have a very strong competitive impact, unlike other scenarios. In other words, competition is here globally asymmetric, in the sense that it decays with trait difference in one direction only, whereas competition is locally asymmetric in the body size model, in which competition asymmetry vanishes for different-enough species.

	**Niche**	**Body size**	**LH trade-off**
g function	Lotka-Volterra	Lotka-Volterra	Lotka-Volterra
Spatial structure?	No	No	Yes (implicit)
Evolving trait	Niche position	Body size	Colonization rate
Viability domain	$(-\infty, \infty)$	$(0, \infty)$	(μ, ∞)
Competition	symmetric	Locally asymmetric	Globally asymmetric
Interference competition	No	Yes	Yes
Argument to a function	$x_i - x_j$	$x_i - x_j$	(x_i, x_j)
One-species k-selection	+/-	+/-	+
One-species a-selection	0	+	-
One-species radiation*	Possible	Possible	Impossible
Alternative evolutionary attractors*	Yes	No	No
Runaway evolution*	Impossible	Possible	Impossible

* *within the context of the functional forms we have studied.*

Supplementary Table 1. An overview of the three ecological scenarios considered.

A complete presentation of the evolutionary dynamics of these models would be out of place in this article, but a brief overview of the similarities and differences between these three ecological scenarios is presented in Supplementary Table 1. Parameter values are summarized in Supplementary Table 2.

	**Niche**	**Body size**	**LH trade-off**
Parameters	s_k and s_a	s_a and d	α and γ
Range	$(0.75, 1.25)$ and $(0.75, 1.25)$	$(1.2, 2.5)$ and $(0.08, 0.32)$	$(2, 15)$ and $(0.4, 1)$
Other parameters	-	-	$\mu = 0.1$

Supplementary Table 2. Bifurcation parameters and their range.

Supplementary Note 2

From equation (2) in the main article, we define fitness as $s(x_m)$, the initial rate of increase of a rare variant with trait value x_m

$$s(x_m) = r(x_m) g\left(\frac{k(x_m)}{\sum_{j=1}^{s} a(x_m, x_j) n_j}\right) \tag{16}$$

In this expression the abundance of resident species n_j are at their resident equilibrium value determined from eq. (2) in the main article; they can thus be treated as independent of x_m considering the mutant to be rare intially.

First-order analysis (directional evolution)

The selection gradient on each species trait is obtained as:

$$\begin{aligned} s'(x_m) = r(x_m) g'\left(\frac{k(x_m)}{\sum_j a(x_m, x_j) n_j}\right) \left(\frac{1}{\sum_j a(x_m, x_j) n_j} \frac{dk}{dx_m} - \frac{k(x_m)}{\left[\sum_j a(x_m, x_j) n_j\right]^2} \sum_j n_j \frac{\partial a(x_m, x_j)}{\partial x_m}\right) \\ + \frac{dr}{dx_m} g\left(\frac{k(x_m)}{\sum_j a(x_m, x_j) n_j}\right) \end{aligned} \tag{17}$$

evaluated at $x_m = x_i$ for every focal species i.

Since at an ecological steady state equation (2) in the main text must be set to zero for all species, we have

$$\frac{k(x_m)}{\sum_j a(x_m, x_j) n_j} = 1 \tag{18}$$

and thus

$$g\left(\frac{k(x_m)}{\sum_j a(x_m, x_j) n_j}\right) = 0 \tag{19}$$

Equation (17) can thus be simplified as

$$s'(x_m) = \frac{r(x_m)}{k(x_m)} g'(1) \left(\frac{dk}{dx_m} - \sum_j n_j \frac{\partial a(x_m, x_j)}{\partial x_m}\right) \tag{20}$$

Any selection on the intrinsic growth rate (r) has canceled out, owing to the assumption that we are close to ecological equilibrium.

All usual g functions (and all those considered here) are strictly increasing so that

$$g'(1) > 0 \tag{21}$$

Hence the sign of the selection gradient is that of the parenthesis in equation (20).

Numerical continuation and fitness curvature

We approximated the evolutionary dynamics of species traits as a gradient-ascent process based on the selection gradient $s'(x_m)$. Specifically we considered the so-called canonical adaptive dynamics equations

$$\frac{dx_i}{dt} = \nu n_i s'(x_i) \tag{22}$$

to determine the (directional) coevolution of trait values. Here ν is a parameter controlling the rate of evolution, which we set to one for all species.

For each ecological scenario, we derived the analytical expression of equation (20) so as to be able to compute the selection gradient of each species in a given community without resorting to numerical differentiation. Equilibrium species abundances n_j were obtained numerically as the null-space of the linear system defined by equation (2) in the main text.

We identified all convergence stable evolutionary singular coalitions for one, two, three, four and five species, by randomly screening the set of possible initial conditions (trait values) and iterating equation (22) until convergence. Once the different evolutionary attractors were identified, for each diversity level and ecological scenario, we followed their position continuously through the entire parameter space using a continuation approach (the parameters varied and the range of variation are given in Supplementary Note 1). In the niche scenario, alternative evolutionary attractors could coexist, differing in their symmetry properties. In the other scenarios, only one attractor could exist per diversity level and parameter values. During the continuation algorithm, we recorded the position (i.e. species trait values) of the attractors, and their potential disappearance or loss of convergence stability. We also numerically computed the curvature of the fitness landscape around each species to determine the evolutionary stability of the attractor. These computations allowed us to resolve and locate evolutionary bifurcations (changes in the nature and evolutionary stability of evolutionary equilibria) and to compute the fraction of parameter space in which adaptive radiations can occur (Figure 3). All these analyses were conducted in R using functions in packages “RootSolve” and “NumDeriv”.

Second-order analysis (evolutionary stability)

We can proceed as above and take the first derivative of the selection gradient from equation (20). The calculation is a bit tedious but straightforward. Intermediate stages are too lengthy to be printed here, but can be considerably simplified using the fact that we restrict our attention to evolutionary singular coalitions, which implies

$$\frac{dk}{dx_m} - \sum_j n_j \frac{\partial a(x_m, x_j)}{\partial x_m} = 0 \tag{23}$$

It follows that all terms vanish except one and we obtain

$$s''(x_m) = \frac{r(x_m)}{k(x_m)} g'(\cdot) \left(\frac{d^2k}{dx_m^2} - \sum_j n_j \frac{\partial^2 a(x_m, x_j)}{\partial x_m^2} \right) \tag{24}$$

From the same arguments as above, it follows that this has the same sign as the parenthesis. Therefore species i is at an evolutionary stable point if

$$\frac{d^2k}{dx_i^2} < \sum_j n_j \frac{\partial^2 a(x_i, x_j)}{\partial x_i^2} \tag{25}$$

This has a simple interpretation: evolutionary stability is determined by the summed effects of quadratic selection on carrying capacity (l.h.s.) and quadratic selection on competition (r.h.s.). We go one step further and

decompose the effect of competition (r.h.s.) as the product of total abundance (community biomass) and per-capita competitive effect (i.e. average competitive effect per individual). We write total abundance as

$$B = \sum_j n_j \tag{26}$$

, the relative frequency of any species j as

$$p_j = n_j/B \tag{27}$$

and the per-capita competition effect experienced by species i as

$$\langle a(x_i)\rangle = \sum_j p_j a(x_i, x_j) \tag{28}$$

This dissociates two components of total competition pressure: total abundance on the one hand, and per-capita competitive effect on the other hand. The first is a pure mass effect, the second is a pure trait-mediated effect (it depends on the relative distribution of individual traits in the community). We can thus write the evolutionary stability criterion as

$$\frac{d^2k(x_i)}{dx_i^2} - \frac{\partial^2 \langle a(x_i)\rangle}{\partial x_i^2} B < 0 \tag{29}$$

This condition is sufficient to determine the evolutionary stability of a singular coalition of species. If every species in the coalition is a fitness maximum (i.e. eq. (29) is satisfied), then the coalition is evolutionary stable. If at least one species is not at a fitness maximum, then the coalition is evolutionary unstable. In the context of this article, we are only interested in feasible and convergence-stable coalitions of species (i.e. evolutionary attractors), as only those are relevant to adaptive radiations. Considering that a species is at an evolutionary attractor, not being at an ESS generically entails that mutual invasibility is possible in its neighborhood, and effectively means that the species is at a branching point, where diversification is possible. Requiring eq. (29) to be violated for at least one species in the community, and introducing the H and I notations as indicated in the main text, yields eq. (1) in the main text.

Remember that all derivatives and quantities should be evaluated at the evolutionary attractor and at the corresponding ecological equilibrium which, following standard practice, is explicitly indicated in the main text by using star superscripts.

Role of components in evolutionary bifurcations

For each ecological scenario, diversity level, and parameter combination, we computed numerically the value of the three components involved in the evolutionary stability criterion (H and I for every species, and B). The raw values of the components are presented in Supplementary Figure 1. We then located all evolutionary bifurcations corresponding to a loss of evolutionary stability when one species was added, i.e. a case where a coalition of s species was evolutionary stable (no radiation) but the coalition of $s+1$ species was evolutionary unstable (radiation possible). For the niche and body size scenarios, we had $1 \to 2$, $2 \to 3$ and $3 \to 4$ bifurcations, meaning that adaptive radiations were triggered by adding one species to communities of one, two and three species, respectively. For the LH trade-off scenario, we only observed the first two kinds of bifurcations (as radiations occured for all parameter combinations in three species communities). For each evolutionary bifurcation, we compared the stability criterion for s species

$$crit_s = H_{i_s} - I_{i_s} B_s < 0 \tag{30}$$

and the criterion for $s+1$ species

$$crit_{s+1} = H_{i_{s+1}} - I_{i_{s+1}} B_{s+1} > 0 \tag{31}$$

where i_s and i_{s+1} represent the index of the species yielding $crit_s$ and $crit_{s+1}$, respectively (see eq. (1) in main text).

Evolutionary bifurcations occur when $crit$ changes sign. A bifurcation therefore requires that the difference $\Delta = crit_{s+1} - crit_s$ be sufficiently positive. This variation in $crit$ is caused by the variations of the different evolutionary components. We therefore computed the change in $crit$ that would be caused by the variation of each component, individually, by varying each component one at a time to obtain theoretical criterion values

$$crit_H = H_{i_{s+1}} - I_{i_s} B_s \quad crit_I = H_{i_s} - I_{i_{s+1}} B_s \quad \text{and} \quad crit_B = H_{i_s} - I_{i_s} B_{s+1} \tag{32}$$

and expressed the corresponding variations, relative to the actual variation:

$$\Delta_H = (crit_H - crit_s)/\Delta \quad \Delta_I = (crit_I - crit_s)/\Delta \quad \text{and} \quad \Delta_B = (crit_B - crit_s)/\Delta \tag{33}$$

These values were then averaged and reported in Figure 5 of the main article.

Supplementary Note 3

A detailed account of evolutionary trajectories and equilibria for each of the three ecological scenarios, as we obtained using the general methods presented in the previous section, is well beyond the scope of this article. However, to provide an illustration, we present here detailed results for the case that received most attention in the literature: the niche scenario (symmetric competition).

One-species dynamics

Irrespective of the choice of functions for k and a, a single species will always evolve to a singular strategy that maximizes the carrying capacity, i.e its trait evolves to $x = 0$ (see Figure 2a in the main document). This singular strategy may either be an ESS, or a branching point, depending on the relative widths of the competition and carrying-capacity functions (see Figure 3a in the main document). This evolutionary behavior is well-known and will not be described any further (see [7;4;8]).

Two-species dynamics

Things become more complicated when considering a diversity of $s = 2$ species. The historical assumption is that both k and a are Gaussian functions, differing in their respective widths (standard deviations) s_k and s_a. In that case, it is predicted that if the one-species singular strategy is a fitness minimum (branching point), there exists a symmetric two-species singular coalition. On the contrary, if the one-species singular strategy is a fitness maximum (ESS), there exists no singular coalition. Whereas two species can perfectly coexist ecologically, provided they are sufficiently dissimilar, no combination of species can persist on an evolutionary timescale. The two species invariably converge to the resource maximum $x = 0$, and one of them necessarily goes extinct in the process, restoring the level of diversity to one. In other words, no diversity can be maintained on the long term, evolution systematically eroding diversity back to one. This precludes, of course, any adaptive radiation. These evolutionary predictions can be summarized using a bifurcation diagram (see [9]), in which the location and convergence stability of singular coalitions (see previous section for methods) is plotted as the width of the competition function (s_a) is increased (Supplementary Figure 3).

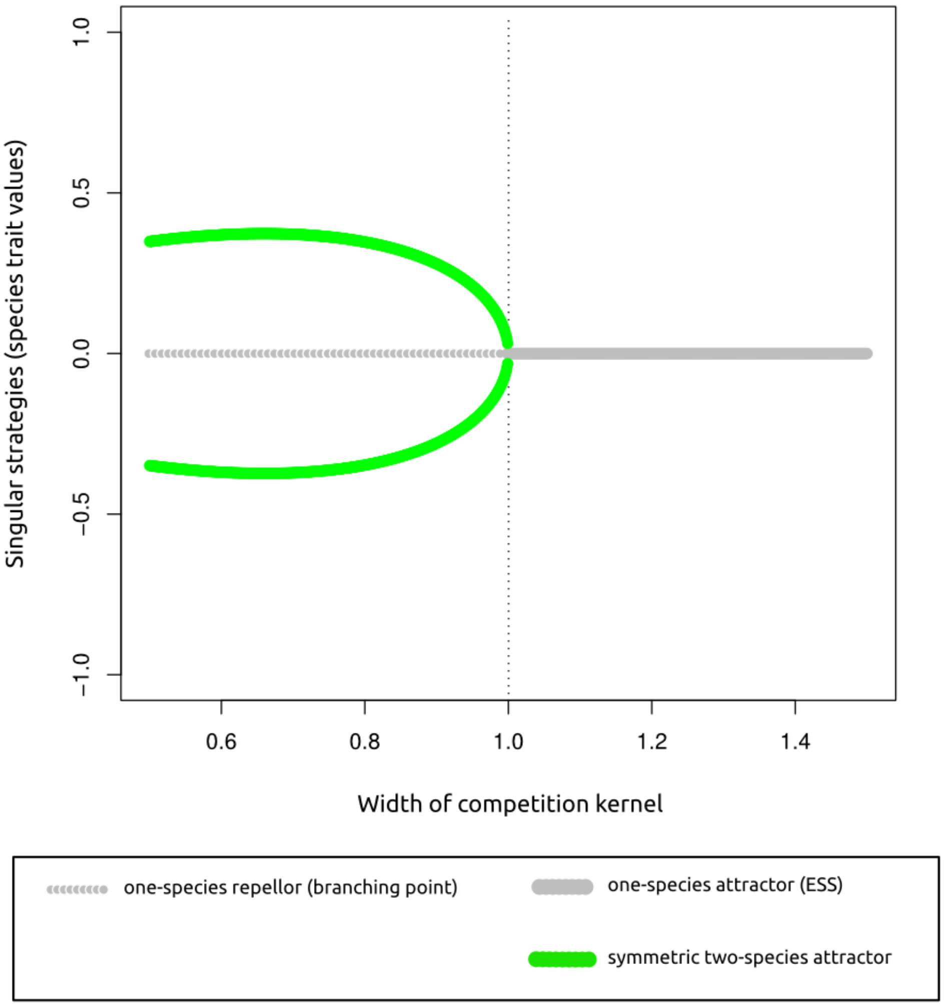

Supplementary Figure 3. Two-species evolution in the historical Gaussian/Gaussian case for the niche scenario. The graph is a bifurcation diagram showing the location (y-axis) and convergence stability (thickness) of all existing evolutionary singularities (see insert) as the width of the competition kernel (s_a) increases (x-axis). In this case one bifurcation (supercritical pitchfork) is observed, when s_a crosses the value of $s_k = 1$ (dotted vertical line). For lower values of s_a the one-species singularity is a branching point and two-species trajectories converge to a symmetric singular coalition. For greater values of s_a, the one-species singularity is an attractor and no two-species singularity exists: all two-species trajectories are absorbed to the one-species attractor.

The impossibility of evolutionary coexistence is, however, an atypical property resulting from this specific choice of functions. Other predictions obtained with this combination of functions were previously shown to be non robust to minor perturbations (e.g.[10]). We here show that the evolutionary prediction that two species cannot coexist on an evolutionary timescale when the one-species singular strategy is an ESS is also non-robust to minor changes in the model assumptions. In fact, the absence of singular coalitions is a degenerate property caused by the assumption of two Gaussian functions for a and k. For other choices of functions, much richer evolutionary dynamics, and predictions more in line with biological intuition, are generically obtained. This is the case with the

functions retained in the main text (Lorentzian k and Gaussian a; eq. (1) and (3)), where evolutionary coexistence of species is possible despite having one-species ESS (see Figure 2a and Figure 3a). To illustrate that this type of evolutionary behavior is not restricted to our choice of functions, we will here extend the definition of the competition function and use:

$$a(x_j, x_j) = \exp\left(-(1-\epsilon)\frac{(x_i - x_j)^2}{s_a^2} - \epsilon\frac{(x_i - x_j)^4}{s_a^4}\right) \tag{34}$$

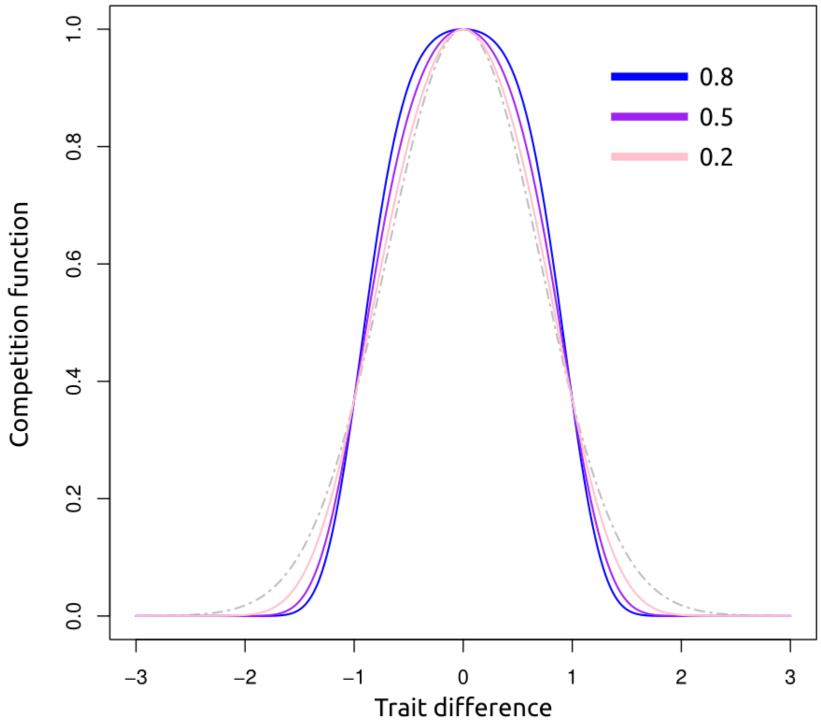

Supplementary Figure 4. General competition function used in section 4 for the niche scenario (eq. (34)). The function is drawn for $s_a = 1$ and different values of ϵ (see legend). For reference the Gaussian function ($\epsilon = 0$) is also shown (gray dashed curve).

This is a generalization of the historical Gaussian function, introducing some degree of a quartic function, through parameter ϵ. If $\epsilon = 0$ we recover the Gaussian a with width s_a (used in the main text), whereas if $\epsilon = 1$, we obtain a quartic function, whose width is similarly governed by the scale parameter s_a. Increasing ϵ from zero thus gradually makes the Gaussian function less peaked (more platykurtic; see Supplementary Figure 4). This function has the advantage, compared to pure generalized Gaussian functions ($\exp(-|x_i - x_j|^z / s_a^z)$ with $z \geq 2$), of having well-behaved first and second-derivatives. As can be seen from Supplementary Fig. 4, the deviation from a purely Gaussian function remains quite small for the values of ϵ tested.

As before, we will summarize the evolutionary dynamics through bifurcation diagrams, plotting the location and stability of all evolutionary singular coalitions of species, as the width of the competition function (parameter s_a) is varied. For different values of ϵ in eq. (34), we obtain similar bifurcation diagrams (see Suppplementary Figures 5 and 6). We also obtain the same type of bifurcation diagrams using a Gaussian carrying capacity instead of eq. (1) (not shown). We thus suggest the evolutionary behavior of the Gaussian/Gaussian case (Supplementary

Fig. 3) is an atypical, degenerate, situation for the niche scenario. Again, our goal here is not to be exhaustive but to establish the possibility of a certain class of evolutionary dynamics.

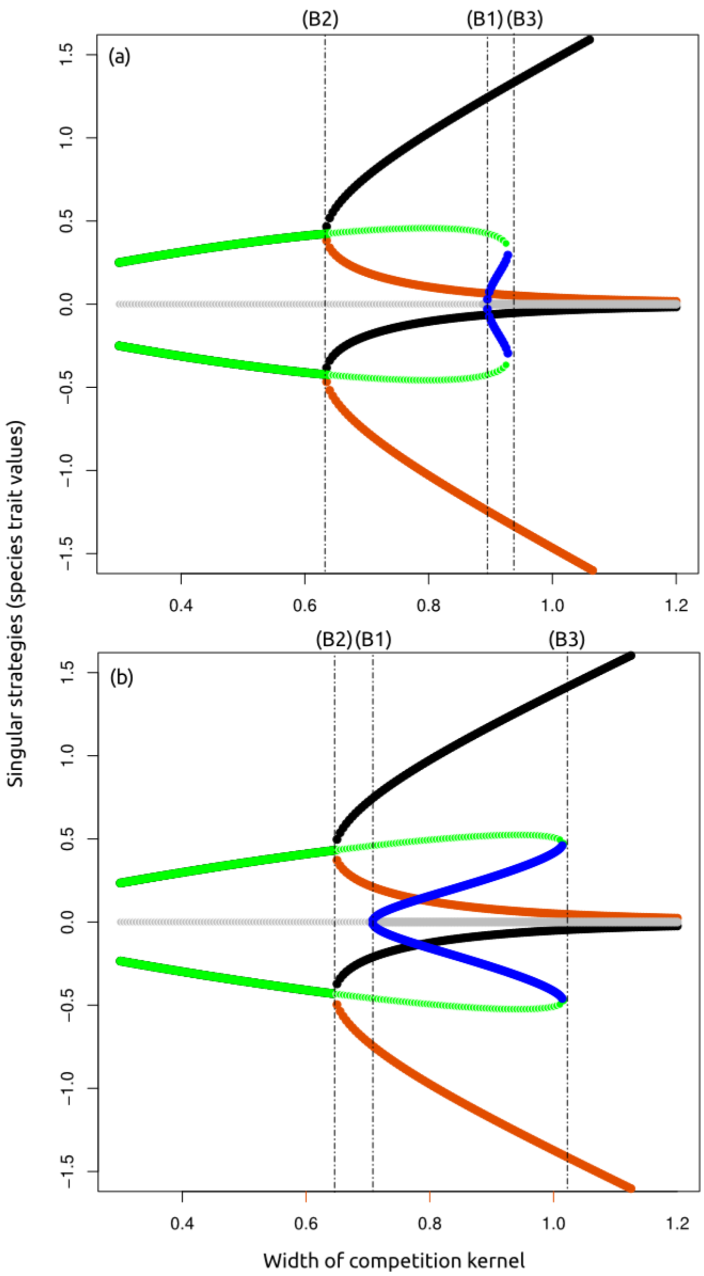

Supplementary Figure 5. Two-species evolution in the niche scenario. Bifurcation diagram showing the location (y-axis) and convergence stability (thickness) of all existing evolutionary singularities as the width of the competition function (s_a) increases (x-axis), using eq. (1)with $s_k = 1$ and eq. (34) with $\epsilon = 0.2$ (a) or $\epsilon = 0.5$ (b). Three bifurcations are observed (dotted vertical lines). See legend panel in Supplementary Figure 6 for a description of all singularites and of bifurcations.

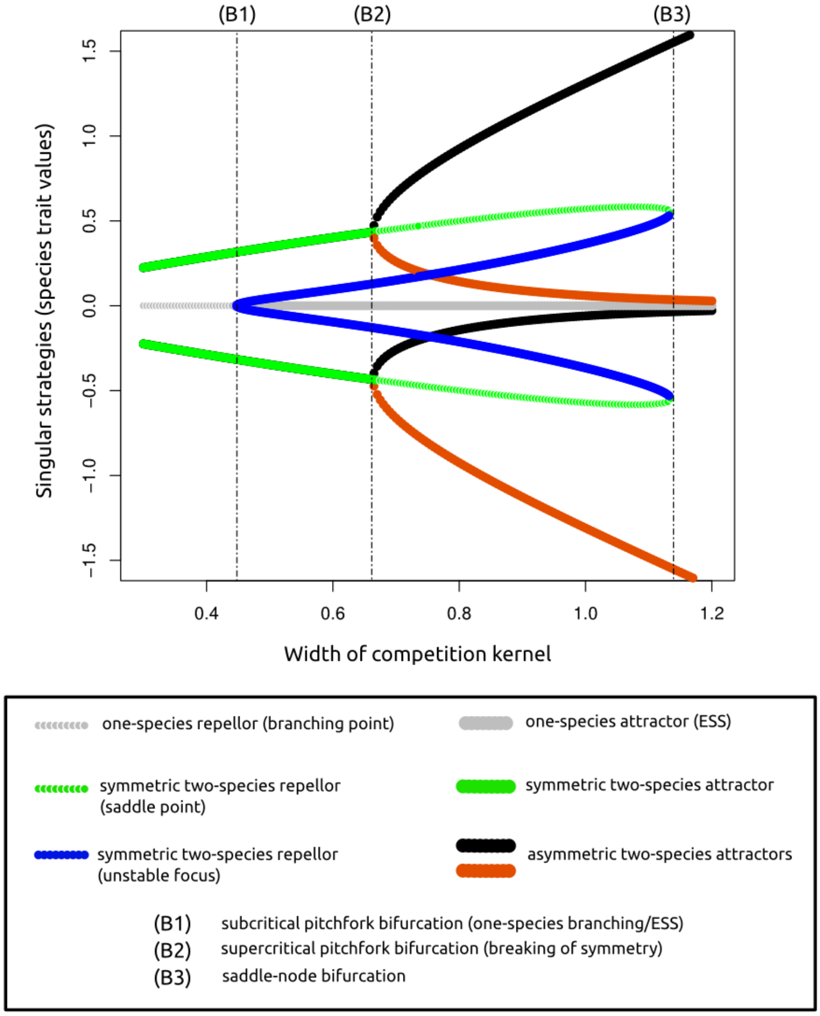

Supplementary Figure 6. Same as Supplementary Figure 5 but with parameter $\epsilon = 0.8$. Three bifurcations are observed (dotted vertical lines). The different singularities and bifurcations observed in Supplementary Fig. 5 and 6 are described in the legend panel.

Compared to the Gaussian/Gaussian case (Supplementary Fig. 3), three different bifurcations are observed and the number of two-species singular coalitions is much greater. Qualitative evolutionary predictions differ importantly. In particular, it is always possible to have evolutionary coexistence of two-species when the one-species attractor is an ESS. This occurs after bifurcation (B1), corresponding to the one-species branching point becoming an ESS. This change occurs as a subcritical pitchfork bifurcation, so that the one-species ESS becomes surrounded by a two-species symmetric repellor (Supplementary Figures 5 and 6; unstable focus in blue). As a consequence, even though the one-species ESS is an evolutionary attractor, its basin of attraction is quite narrow. In most cases, two species will be attracted to another, dimorphic, singular coalition. This behavior corresponds to an evolutionary "limit to similarity", echoing the familiar ecological concept of a limit to similarity: if two species are too similar, evolution will eventually doom one of them to extinction, even though they were able to persist on an ecological timescale. However, if two species are sufficiently dissimilar initially, they can coexist in the long

term.

Furthermore, evolutionary coexistence of two species can occur in two different ways:

- First, the two species can evolve to a symmetric singular coalition, where each is equally distant from the resource maximum ($x = 0$). This was the only possibility in the historical Gaussian/Gaussian case (Supplementary Fig. 3), and is expected for sufficiently narrow competition functions (left of bifurcation (B2); Supplementary Fig. 5 and 6).

- Second, there can be a breaking of symmetry. As the width of the competition function increases, asymmetric two-species evolutionary attractors appear (bifurcation (B2)) and the symmetric two-species attractor becomes a repellor (an unstable manifold for directional evolution) or ultimately disappears entirely (bifurcation (B3)). In these cases, two species can coexist on an evolutionary timescale, but they will not distribute themselves symmetrically on each side of the resource maximum ($x = 0$). Instead, one species will evolve close to the maximum, whereas the second will be maintained further away from it (Supplementary Figures 5 and 6). Intuitively, this represents a form of resource preemption[6;11], by which the species closest to the resource optimum monopolizes it and stands its ground, while the other species is prevented from displacing it. Interestingly, asymmetric evolutionary equilibria appear even though all elements of the model are perfectly symmetric.

The relative position of bifurcations (B1) and (B2) can vary with the shape of the competition function (parameter ϵ). Therefore, depending on the shape of the competition function, we may have symmetric or symmetric coexistence of two-species, regardless of whether the one-species singular strategy is a branching point or not. The only situation where evolutionary predictions overlap with the historical Gaussian/Gaussian case is thus for very narrow competition functions (before the first bifurcation occurs, which can be either (B1) or (B2), depending on ϵ).

Evolutionary dynamics with two species can be best understood using 2D flow diagrams[9]. For any pair of species, the flow diagram shows the sign of the selection gradient for the two species (also called "invasion cones", indicating the direction of evolution;[12]). Such flow diagrams are provided in Figures S7 and S8, representative of the contrasted sections in the bifurcation diagram (Supplementary Fig. 6).

Multiple-species dynamics

With more than two-species, the number of evolutionary attractors increases correspondingly. The attractors can have varying levels of symmetry, from entirely symmetric to completely unbalanced. The existence of the different attractors and their respective basins of attractions depend on parameter values, as in the two-species case. Furthermore, different attractors can coexist for a given set of parameters, which introduces a dependence of evolutionary outcomes on initial conditions (initial trait values of species). This is too complex too be presented here but explains why, in Figure 3a, it is indicated that the result depends on historical factors for three or four species, since different alternative attractors sometimes differ in their evolutionary stability (ESS or branching point).

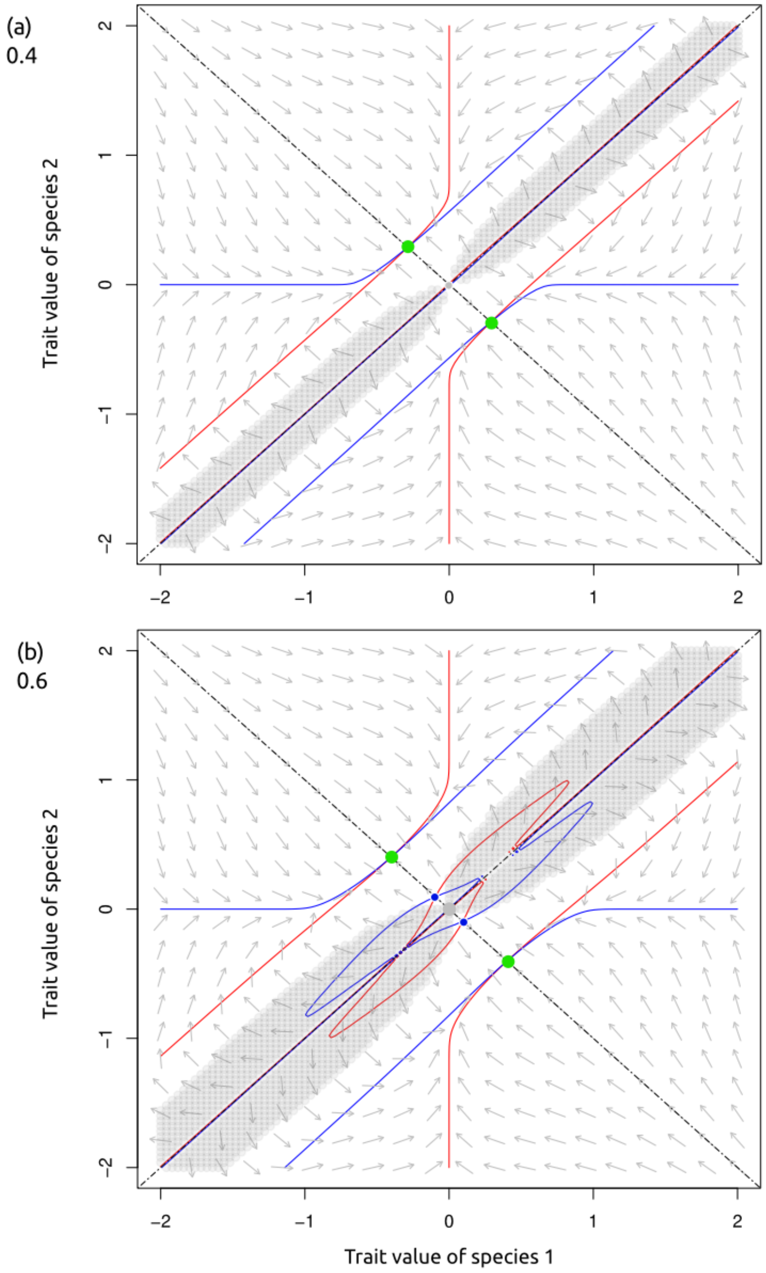

Supplementary Figure 7. Flow diagrams showing the direction of evolution (arrows) when two species are coevolving. Evolutionary singularities are shown as dots, with the same color codes as in the bifurcation diagram (Supplementary Fig. 6). The red and blue curves are the evolutionary isoclines of the two species (values such that the selection gradient annihilates for each species). The gray area indicates combinations of species that cannot coexist ecologically (one drives the other to extinction owing to competitive exclusion). Two-species coevolution can thus only occur in the white area (the so-called area of coexistence). $\epsilon = 0.8$ (as in Supplementary Fig. 6) and (a) $s_a = 0.4$ (b) $s_a = 0.6$.

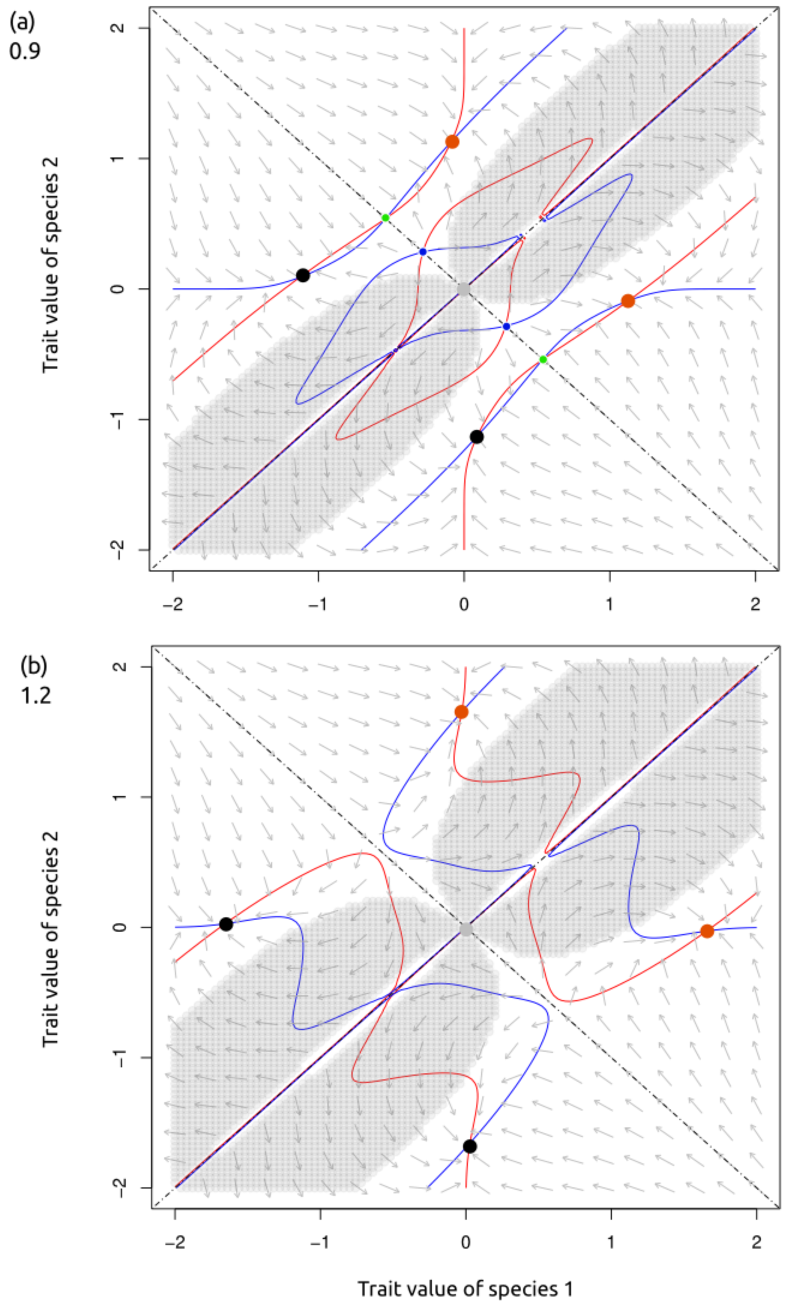

Supplementary Figure 8. Same as Supplementary Figure 7, with (a) $s_a = 0.9$ and (b) $s_a = 1.2$.

Supplementary References

[1] Mahler, D. L., Ingram, T., Revell, L. J. & Losos, J. B. Exceptional convergence on the macroevolutionary landscape in island lizard radiations. *Science* **341**, 292–295 (2013).

[2] Scheffer, M. & van Nes, E. H. Self-organized similarity, the evolutionary emergence of groups of similar species. *Proceedings of the National Academy of Sciences USA* **103**, 6230–6235 (2006).

[3] Rummel, J. & Roughgarden, J. A theory of faunal buildup for competition communities. *Evolution* **39**, 1099–1033 (1985).

[4] Doebeli, M. & Dieckmann, U. Evolutionary branching and sympatric speciation caused by different types of ecological interactions. *The American Naturalist* **156**, S77–S101 (2000).

[5] Tilman, D. Competition and biodiversity in spatially structured habitats. *Ecology* **75**, 2–16 (1994).

[6] Calcagno, V., Mouquet, N., Jarne, P. & David, P. Coexistence in a metacommunity: the competition-colonization trade-off is not dead. *Ecology Letters* **9**, 897–907 (2006).

[7] Dieckmann, U. & Doebeli, M. On the origin of species by sympatric speciation. *Nature* **400**, 354–357 (1999).

[8] Doebeli, M. & Ispolatov, I. Complexity and diversity. *Science* **328**, 494–497 (2010).

[9] Dercole, F. & Rinaldi, S. *Analysis of evolutionary processes: the adaptive dynamics approach and its applications* (Princeton University Press, 2008).

[10] Meszéna, G., Gyllenberg, M., Pásztor, L. & Metz, J. A. Competitive exclusion and limiting similarity: a unified theory. *Theoretical Population Biology* **69**, 68–87 (2006).

[11] Fukami, T. Historical contingency in community assembly: integrating niches, species pools, and priority effects. *Annual Review of Ecology, Evolution, and Systematics* **46**, 1 (2015).

[12] Kisdi, E. Evolutionary branching under asymmetric competition. *Journal of Theoretical Biology* **197**, 149–162 (1999).
